# US military veterans’ perceived concordance with their providers regarding persistent physical symptoms prospectively predicts satisfaction with care and adherence to care plans

**DOI:** 10.1093/abm/kaaf028

**Published:** 2025-04-21

**Authors:** L Alison Phillips, Laura M Lesnewich, Katharine Bloeser, Yong Lin, Rachel L Boska, Justeen K Hyde, Peter J Bayley, Helena K Chandler, Matthew J Reinhard, Susan L Santos, Rachel Stewart, Drew A Helmer, Lisa M McAndrew

**Affiliations:** Department of Psychology, Iowa State University, Ames, IA 50010, United States; War Related Illness and Injury Study Center (WRIISC), Veterans Affairs New Jersey Health Care System, East Orange, NJ 07018, United States; War Related Illness and Injury Study Center (WRIISC), Veterans Affairs New Jersey Health Care System, East Orange, NJ 07018, United States; War Related Illness and Injury Study Center (WRIISC), Veterans Affairs New Jersey Health Care System, East Orange, NJ 07018, United States; Department of Biostatistics and Epidemiology, School of Public Health, Rutgers University, Piscataway, NJ 08854, United States; War Related Illness and Injury Study Center (WRIISC), Veterans Affairs New Jersey Health Care System, East Orange, NJ 07018, United States; Center for Health Optimization and Implementation Research, VA Bedford Healthcare System, Bedford, MA 01730, United States; General Internal Medicine, Chobanian & Avedesian School of Medicine, Boston University, Boston, MA 02118, United States; War Related Illness and Injury Study Center (WRIISC), VA Palo Alto Health Care System, Palo Alto, CA 94304, United States; Department of Psychiatry and Behavioral Sciences, Stanford University School of Medicine, Stanford, CA 94305, United States; War Related Illness and Injury Study Center (WRIISC), Veterans Affairs New Jersey Health Care System, East Orange, NJ 07018, United States; Department of Psychiatry, Georgetown University Medical School, Washington, DC 20037, United States; War Related Illness and Injury Study Center (WRIISC), Veterans Affairs New Jersey Health Care System, East Orange, NJ 07018, United States; War Related Illness and Injury Study Center (WRIISC-DC), Washington DC Veterans Affairs Medical Center, Washington, DC 20422, United States; Center for Innovations in Quality, Effectiveness, and Safety (IQuESt), Michael E. DeBakey VA Medical Center, Houston, TX 77021, United States; Department of Medicine, Baylor College of Medicine, Houston, TX 77030, United States; War Related Illness and Injury Study Center (WRIISC), Veterans Affairs New Jersey Health Care System, East Orange, NJ 07018, United States

**Keywords:** Gulf War illness, persistent physical symptoms, illness perceptions, concordance, adherence, veteran

## Abstract

**Background:**

Medically unexplained, persistent physical symptoms and syndromes are commonly seen in primary care. These are debilitating for patients and difficult to treat, causing frustration for patients and providers.

**Purpose:**

This study investigates how well US military veterans with multiple persistent physical symptoms (PPS), called Gulf War illness (GWI), agree with their healthcare providers about their illness. This agreement, called perceived concordance, is hypothesized to influence veterans’ satisfaction with care, adherence to care plans, and disability levels.

**Methods:**

Participants were 230 veterans with GWI deployed to the 1990-1991 Gulf War who were recruited from Veteran Affairs primary care and War Related Illness and Injury Study Centers (WRIISCs). Veterans’ GWI perceptions and perceived concordance with their providers regarding GWI were assessed at a medical visit. Veterans’ self-reported satisfaction with care, adherence to care plans, and disability levels were measured at 1 week, 1 month, and 6 months post-baseline.

**Results:**

Bivariate correlations indicated that veterans’ GWI-related illness perceptions were related to veterans’ satisfaction with care and reports of functional disability. Beyond these effects, veterans’ perceived concordance with the provider regarding GWI was positively associated with satisfaction over time (e.g., fixed-effect estimate = 0.36, *P* < .001 for 1-week follow-up) and adherence to care plans (fixed-effect estimate averaged across all timepoints = 0.03, *P* = .03) but was unrelated to reported disability.

**Conclusions:**

Veterans’ perceived concordance with their providers about GWI seems to be important for patient satisfaction and adherence to care plans. More research with longer-term follow-up is needed to understand how perceived concordance might influence disability levels and the outcome of care plans.

## Teaser

US Military Veterans’ perceptions about their Gulf War Illness predicted subsequent levels of disability. Beyond these perceptions, Veterans’ perceived concordance with their provider predicted subsequent satisfaction with care and adherence to care plans.

## Introduction

“Medically unexplained” persistent physical symptoms (PPS) and syndromes (commonly co-occurring PPS), such as chronic fatigue, chronic pain, and irritable bowel syndrome are common and debilitating.^1^ These conditions present unique challenges for clinical practice, beyond the patient care challenges presented by common comorbid conditions, such as depression and post-traumatic stress disorder.^2,3^ Recent research has focused on what providers can do to promote treatment adherence and improve management of PPS.^4,5^ The present study addresses these issues and focuses on a particularly important population—military veterans—living with PPS, also termed chronic multisymptom illness (CMI) and Gulf War illness (GWI). GWI is characterized by a heterogeneous set of symptoms in broader symptom domains that include fatigue, pain, neurological/cognitive/mood symptoms, skin-related problems, gastrointestinal issues, and respiratory dysfunction. Upon their return from the 1990-1991 Gulf War, an estimated 25%-30% of Gulf War veterans (GWVs) reported persistent physical symptoms (for months to years post-deployment) in at least 3 of these symptom domains, which could not be explained by other medical or mental health conditions.^6^ GWI is considered an incurable chronic condition that requires lifelong symptom management and is known to be debilitating.^7–9^ Reported levels of disability for GWVs with GWI are more severe than those found among individuals with other severe medical illnesses, like cancer and lung disease.^10–12^

Patients’ understanding of their illness, or illness perceptions, have been proposed as a determinant of outcomes among patients with PPS, as they predict the onset of PPS after an injury or acute illness.^13^ This includes the development of chronic fatigue syndrome after infectious mononucleosis,^14^ post-concussive syndrome after a mild traumatic brain injury,^15^ and irritable bowel syndrome after food poisoning.^16^ Changes in illness perceptions also account for improvements in behavioral treatments for chronic physical symptoms.^17^ Among GWVs with GWI, illness perceptions account for 33% of the variance in physical health functioning levels levels.^18^

As delineated in the Common-Sense Model of Self-Regulation (CSM),^19^ illness perceptions are comprised of identity (e.g., what is the illness and its associated symptoms), timeline (e.g., how long will I have it; does it come and go), cause (e.g., what caused it and what maintains it), consequences (e.g., what will happen to me physically, socially, or financially as a result of the illness), and control (e.g., what can I do to manage the illness). Illness perceptions (alternatively called illness beliefs or representations^20,21^) are part of a complex self-management process in which patients monitor their physical symptoms and well-being, develop illness perceptions, and then manage their illness based on their illness perceptions.^20,22^ Illness perceptions consistently predict health outcomes after accounting for other factors, such as illness severity. For example, after a mild head injury, illness perceptions have predicted with 85% accuracy who will develop PPS, after accounting for the severity of injury, depression, and anxiety.^15,23^

Patients’ illness perceptions guide self-management (i.e., behavior), just as a provider’s understanding of disease guides their treatment recommendations. Illness perceptions determine if patients seek healthcare, take medication, exercise, and adhere to other recommendations,^21,24^ thus influencing health outcomes. For example, a veteran with GWI whose illness perception includes the belief that physical activity exacerbates their GWI may avoid exercise. A veteran with GWI who believes that their GWI is causing heart damage may eat a heart-healthy diet and do aerobic exercise. These self-management behaviors, in conjunction with underlying physiological causes of GWI and the impact of self-management behaviors on the underlying pathophysiology, influence disability levels.^25^

Beyond specific illness perceptions, patients’ degree of perceived *concordance* with their medical provider regarding the domains of illness perceptions (the cause, consequences, associated symptoms and diagnosis, treatment/control, and timeline of GWI) is important for patient’s adherence to GWI-related recommendations and relevant outcomes.^19,26^ Concordance is defined as the negotiated, shared understanding between patients and providers on illness perceptions critical to care.^26,27^ When patients hold an illness representation that runs counter to the medical model of an illness (and therefore is presumably non-concordant with the provider’s representation of the patient’s illness), the negative effect on treatment adherence and health is clear: for example, individuals with asthma who hold an acute timeline belief—that they have asthma only when they have symptoms of asthma—are far less adherent to prophylactic asthma medication and are much more likely to end up in hospital with an asthma attack than patients with a medically accurate, chronic timeline model of asthma.^19^ Perceived concordance with the provider need not align with the medical model to influence adherence and health outcomes positively—indeed, perceived concordance is an important part of the working alliance between patient and provider and may foster adherence to prescribed care plans and continuity of care through optimal trust in the provider.^27^

Research to date has provided some support for the importance of perceived concordance for patient adherence and health outcomes: Patients’ perceived concordance with their medical provider on the presenting symptom(s) and care specifics has been found to predict patients’ reported adherence and problem resolution in a civilian primary care sample.^28^ Perceived concordance between patient and provider is relatively less studied in military veterans and other populations with PPS, but Phillips and McAndrew^29^ found that when military veterans with GWI perceived non-concordance with their provider, regarding the illness and treatment specifics, the veteran had lower levels of satisfaction with the provider and with medical care overall. Research has yet to evaluate the importance of veterans’ perceived concordance regarding their GWI perceptions in predicting GWI treatment adherence and related outcomes over time.

Given the above prior literatures, and based on the Common-Sense Model of Self-Regulation,^19^ we hypothesized that: Veterans with GWI who perceive greater concordance in illness perceptions with their provider (assessed immediately after the medical encounter) will report greater satisfaction with their care from that provider, greater adherence to the provider’s treatment care plans for the veteran, and reduced levels of disability 1 week, 1 month, and 6 months after the encounter. These relationships between perceived concordance and outcomes were expected, even after accounting for the relationship of veterans’ specific GWI perceptions with the outcomes.

## Method

The hypotheses, study design, measures, and analysis plan were determined prior to data collection in the form of an independently reviewed and funded research proposal to the United States Department of Veteran Affairs (VA), available from the corresponding author.

### Procedure

Three VA sites participated in the current study (VA New Jersey Health Care System in East Orange, NJ; VA Palo Alto Health Care System in Palo Alto, CA; and the Washington, DC VA Medical Center), all with primary care clinics and tertiary care sites for veterans with GWI, called the War Related Illness and Injury Center (WRIISC). Veterans with scheduled appointments at these sites were sent introductory recruitment letters detailing the study opportunity and were then called 2 weeks later with an invitation to participate in the study. Screening for eligibility occurred during this phone call. Recruitment, screening, and medical appointments for the study occurred between 2016 and 2019. The total number of individuals approached for recruitment is not known, because we used a broad recruitment strategy via posters, word of mouth, and postal mailings.

Eligibility to participate in the study was confirmed via the computerized patient record system (CPRS) by research staff. More than half of those that were screened were eligible for the study, and over three-quarters of those who screened as eligible enrolled in the study. Eligible volunteers were then sent an information sheet and an initial set of baseline survey measures, including measures of demographics, depressive and Post-Traumatic Stress Disorder (PTSD) symptom experience, illness (GWI) perceptions, information on deployment history to the Gulf War areas, and baseline levels of adherence to treatment care plans and disability, via postal mail. Participants returned the completed baseline surveys prior to their scheduled medical visit, although they had the option to complete the baseline survey at the time of the medical visit, if that was their preference.

An in-person informed consent process took place at the participants’ scheduled medical visit at either the WRIISC or at primary care. Participants completed the remainder of the baseline survey measures, including measures of perceived concordance with the provider and satisfaction with care from the provider, after the scheduled medical visit. They were then asked to complete 3 follow-up surveys, which assessed outcome measures (i.e., satisfaction, adherence, and disability) at 1 week, 1 month, and 6 months post-visit. These follow-up surveys were sent via postal mail and returned via pre-paid postal mail or in person, per the participant’s preference. Participants were sent postal mail reminders and were called by phone to remind them to complete the follow-up surveys. They were also given the option of answering the survey questions over the phone. Survey responses for each follow-up timepoint were accepted until the subsequent follow-up survey was sent out.

Participants were compensated with up to $230. They received $45 for completing the baseline assessments, $40 for completing the 1-week follow-up, $40 for completing the 1-month follow-up, and $75 for completing the 6-month follow-up. All study procedures were approved by the VA Central Institutional Review Board (cIRB).

### Participants

The sample consisted of 237 veterans deployed to the Gulf War who met the criteria for GWI; 7 veterans consented to participate but did not provide any data. Approximately half of the veterans (113; 49.1%) in the study were from the primary care clinics, with 117 (50.9%) from the WRIISCs. There was some attrition over time, with 223 (97%) completing the baseline survey, 197 (85.7%) completing the 1-week survey, 190 (82.6%) completing the 1-month survey, and 167 (72.6%) completing the 6-month survey. Specific reasons for attrition are not known, but completers did not differ from non-completers on demographics or baseline levels of the variables, and the statistical analysis approach used multiple imputations to handle missing values (see *Statistical analyses* in the *Method* section).

We conducted an *a priori* power analysis using PASS 12 from NCSS, Inc., and assumed a 2-sided type I error rate of 0.05 using a repeated measures analysis (based on Geisser–Greenhouse corrected F tests), with 1 between-subjects factor (perceived concordance) and 1 within-subjects factor (time). This design achieves 100% power to test the between factor and the within factor with an effect size (used as an index of the size of the mean differences relative to the SD) of 0.5 for both factors, with a sample size of 200. For effect sizes equal to 0.25 (halving the effect size for both factors), the design would still achieve 90% power.

Veterans were eligible for the current study if they met the Kansas definition of GWI and had been deployed to the Persian Gulf region between August 1990 and July 1991. The Kansas case definition of GWI provides an appropriate, empirically based system of identifying GWVs who continue to suffer from chronic multi-symptom illness.^30^ The Kansas case definition identifies 6 symptom domains and inclusion requires that veterans endorse moderately severe and/or multiple symptoms in at least 3 of those domains. The 6 symptom domains are: fatigue, pain, neurological/cognitive/mood, skin, gastrointestinal, and respiratory. Veterans must also indicate that each of those symptoms first became a problem during or after the Gulf War. Self-reported physician diagnoses of chronic conditions (e.g., cancer) that are not associated with Gulf War service, but that can produce diverse symptoms (e.g., pain) or interfere with respondents’ ability to accurately report their symptoms (e.g., severe cognitive decline) are exclusionary. This definition was derived from a population-based survey of over 2000 Gulf War veterans. As this is an aging population, only veterans with a condition that clearly can account for the symptoms of CMI (e.g., multiple sclerosis) were excluded.

### Measures

#### Veteran illness perceptions

Gulf War illness perceptions were assessed using the Illness Perception Questionnaire, Revised (IPQ-R).^31^ The IPQ-R for GWI was developed to measure the 5 components of illness perception (identity, consequences, timeline, control/cure, and cause), based on the Common-Sense Self-Regulation Model; for example, “My GWI has major consequences on my life.” The IPQ-R is meant to be used flexibly (edited appropriately) to capture patients’ understanding of their condition, in this case, GWI. The IPQ-R has subscales with items on a 5-point Likert-type scale ranging from 1 (*strongly disagree*) to 5 (*strongly agree*). Items from each subscale are summed, after accounting for reverse scores, to calculate the score for each subscale; all but one of the subscales had a possible range of scores from 6 to 30 (from 6 items in each subscale), with the cyclic timeline beliefs subscale having only 4 items and therefore a range of possible scores from 4 to 20. Higher scores on the subscales indicate, respectively: a longer, more chronic timeline (versus a short, acute timeline); greater perceived consequences of GWI; greater perceived control of GWI; greater endorsement of a cyclic (versus stable) experience of symptoms; and more severe influence of GWI on the participant’s mood/emotions. Note that not all CSM domains are represented by a sum of item scores; specifically, cause-related and identity-related beliefs are not represented by an interval variable, because they cannot be quantified on a single quantitative range of scores (e.g., participants report the causes they think led to their illness and they report whether or not symptoms are associated with their illness and/or state the diagnosis/label they perceive to be most accurate for representing their condition). Table 1 provides descriptive statistics of the subscales and correlations between subscales and other variables for the current sample. A full list of the items in each subscale are available on the Open Science Framework (link).

**Table 1.** Descriptive statistics and bivariate correlations.

Variable	Mean (*SD*)	1	2	3	4	5	6
1. IPQ-R Chronic Timeline Beliefs	27.19 (3.66)						
2. IPQ-R Consequences Beliefs	24.93 (4.03)	0.65**					
3. IPQ-R Personal Control Beliefs	17.91 (4.36)	−0.27**	−0.29**				
4. IPQ-R Cyclic Timeline Beliefs	13.76 (3.59)	0.04	0.09	0.01			
5. IPQ-R Emotional Representation	20.81 (3.32)	0.34**	0.53**	−0.28**	0.23**		
6. Perceived Concordance	21.01 (7.25)	0.16*	0.21*	−0.03	−0.11	0.03	
Satisfaction, Prior-Visit Baseline	21.57 (4.98)	−0.27**	−0.36**	0.14*	−0.09	−0.16*	−0.10
Satisfaction, 1-week	26.89 (4.98)	−0.02	−0.08	0.18*	−0.07	−0.12	0.40**
Satisfaction, 1-month	26.40 (4.96)	−0.02	−0.08	0.11	−0.10	−0.16*	0.37**
Satisfaction, 6-months	26.34 (4.92)	−0.07	−0.11	0.14	−0.01	−0.11	0.30**
Adherence, Prior-Visit Baseline	4.22 (0.98)	0.09	−0.01	0.03	−0.13	−0.12	0.11
Adherence, 1-week	3.96 (1.06)	−0.12	−0.11	0.04	−0.04	−0.12	0.17*
Adherence, 1-month	4.02 (1.11)	−0.08	−0.07	−0.04	−0.10	−0.04	0.04
Adherence, 6-months	4.10 (1.05)	0.02	−0.02	0.03	−0.15	−0.10	0.19*
Disability, Prior-Visit Baseline	50.99 (18.70)	0.25**	0.51**	−0.35**	0.07	0.43**	0.08
Disability, 1-week	49.88 (19.78)	0.22*	0.48**	−0.26**	0.001	0.34**	-0.03
Disability, 1-month	49.38 (19.84)	0.28**	0.45**	−0.22*	0.03	0.30**	0.05
Disability, 6-months	51.17 (21.16)	0.23*	0.50**	−0.31**	0.01	0.38**	0.01

Note: Correlations are provided between all illness perception variables from the IPQ-R and perceived concordance. Correlations are also provided between these variables and the outcomes at all timepoints. Significance levels are noted by: *P < .05, **P < .001. Satisfaction with care was measured via the PSQ-18. Disability was measured via the WHO-DAS 2.0. Adherence to GWI care plans was measured via the GAS.

#### Concordance of illness perceptions

Concordance of illness perceptions was measured via the following 6 items, originally developed by Phillips et al.^28,29^ in a primary care setting and adapted for the current study to be about GWI: *(1) The provider’s view of how long my GWI will last is similar to my view. (2) The provider and I agree on the cause of my GWI. (3) The provider and I seemed to agree on my ability to influence my GWI. (4) The provider and I agree on how my GWI affects my life. (5) Overall, the provider and I share a common understanding of my GWI. (6) Overall, the provider and I share a common understanding of the treatment for my GWI.* The response options are on a 5-point Likert-type scale, with 1 = not at all and 5 = very much, and responses were summed to create the scale variable (possible range from 6 to 30), with greater values indicating greater perceived concordance. Baseline values (assessed immediately after the medical encounter) were used in the current analyses. Cronbach’s alpha for the current sample at baseline was 0.95.

#### Treatment adherence

Treatment adherence was assessed using the General Adherence Scale (GAS). The GAS is a 5-item self-report measure of general adherence to medical recommendations in the previous 4 weeks^32^ and was initially developed for the Medical Outcomes Study.^33^ A general measure was chosen, versus treatment-specific, to be relevant to all veterans’ experiences, as treatment options for GWI are highly varied. The responses are on a 6-point Likert-type scale (*None of the time, A little of the time, Some of the time, A good bit of the time, Most of the time, All of the time*), with the total score obtained by averaging responses to the 5 items (possible range from 1 to 6, with higher scores indicating greater adherence): *(1) I had a hard time doing what my provider suggested I do. (2) I followed my provider’s suggestions exactly. (3) I was unable to do what my provider’s treatment plans suggested. (4) I found it easy to do the things my provider suggested I do. (5) Generally speaking, how often during the past 4 weeks were you able to do what the provider told you?* The scale has adequate internal consistency (α = .81), with a test-retest reliability of.40. The GAS had a low correlation (*r* = .15) with a social desirability scale, indicating that the measure is not significantly susceptible to social desirability bias.^34^ Cronbach’s alpha for the current sample was 0.70.

#### Satisfaction

Satisfaction was measured using the Patient Satisfaction Questionnaire-18, an 18-item self-report measure with 7 subscales (general satisfaction, technical quality, interpersonal care, communication, financial aspects, time spent, and access/availability/convenience) and a composite score that can be used to measure both visit specific and general satisfaction; the measure is a valid and reliable measure of patient satisfaction.^35^ The PSQ is a widely used measure of patient satisfaction in healthcare services^36^ and has been used specifically to assess patient satisfaction of VA healthcare services among chronically ill veterans.^37^ The measure asked about the veteran’s experience with their WRIISC or primary care provider, depending on where they were recruited. We used a sum of the subscale scores as the composite (range 8-34.5), with higher values indicating greater satisfaction with their care by the provider. Cronbach’s alpha for the current sample was 0.91.

#### Disability

The World Health Organization Disability Assessment Schedule 2.0 (WHO-DAS 2.0^38–40^) measures disability due to physical and mental health conditions. The WHO-DAS 2.0 is a 40-item measure that assesses the degree to which functioning is affected in 6 life task domains: Understanding and communicating; Self-care; Mobility (getting around); Interpersonal relationships (getting along with others); Work and household roles (life activities); and Community and civic roles (participation). We used the composite score, which is calculated via a published algorithm^40^ that combines the 6 subscale scores, as a dependent variable in the present study. The WHODAS 2.0 is based on the International Classification of Functioning, Disability and Health (ICFDH-2). The items of the WHODAS 2.0 have a factor loading on composite score of 0.82-0.98, and the possible range of scores is 0-100, with higher values indicating greater experienced disability. Cronbach’s alpha in this sample was 0.94.

#### Post-traumatic stress disorder symptoms

The PTSD Checklist—5 (PCL-5) is a 20-item National Center for PTSD Checklist used to assess PTSD symptoms (range 0-80).^41^ The PCL-5 asks Veterans how much they have been bothered by a particular symptom over the last month. The PCL-5 has been shown to be a valid and reliable measure of PTSD symptoms.^39^ The PCL-5 has been previously used in studies with GWVs^41–43^ and was assessed as a possible confounder in the current analyses, since PTSD experience is common in Veterans and can influence reports of the outcomes of interest.^41–43^

#### Depressive symptoms

The Patient Health Questionnaire-8 (PHQ-8) is an 8-item self-report questionnaire that assesses the frequency of depressive symptoms over the past 2 weeks. The total score of the PHQ-8 has also been used as indicative of depression severity (range 0-27). The PHQ-8 has been previously used in studies with GWVs^44,45^ and was assessed as a possible confounder in the current analyses, since depression is common among veterans and can influence reports of the outcomes of interest.^44,45^

### Statistical analyses

Missing data were imputed using the fully conditional specification (FCS) multiple imputation approach, assuming missing at random. Twenty-five imputed datasets were generated based on age, sex, visit type, PTSD symptoms, and depressive symptoms. Statistical analysis was performed on each imputed dataset and combined using Rubin’s rule via Proc MI and Proc Mianalyze in SAS. For all analyses, a 2-sided *P* value < .05 was considered statistically significant. All analyses were performed using SAS Enterprise Guide Version 8.3 (SAS Institute Inc, Cary, NC).

Before testing the hypothesis regarding the relationship between concordance and outcomes over time, we evaluated bivariate relationships between illness perceptions, concordance, potential confounders (PTSD symptom burden, depressive symptom severity), and outcomes at all 4 timepoints. To test the hypothesis, we evaluated each patient outcome variable separately using marginal-generalized estimating equations (GEEs) linear models with an individual participant as the clustering unit. The key predictor variables were veterans’ perceived concordance with the provider and the veterans’ GWI perceptions (perception types treated as separate variables), measured at baseline. In the GEE model, time was used as a categorical variable, since the amount of time between follow-up assessments was highly varied. The inclusion of time and its interaction with perceived concordance allowed us to assess if the hypothesized relationship changed over time. Note that although concordance was treated as a continuous variable in analyses, the figures show the differences in outcomes for low, medium, and high concordance values, determined by tertile split on concordance. We expected to find support for the hypothesis in either a main effect of baseline perceived concordance on the outcomes and/or a significant interaction effect between perceived concordance and time in predicting the outcomes (adherence, disability, and satisfaction). All analyses controlled for veterans’ GWI perceptions and the participants’ visit type (WRIISC, primary care only). We conducted all GEE tests with and without PTSD and depressive symptom severity.

## Results

Approximately 48% of the sample were between the ages of 45 and 50, with the other 52% being over the age of 50. Participants identified as Black (26%), Hispanic (9%), White (52%), or multiple or other racial and ethnic identities (12%), and 86.6% identified as men. Supplementary material (link) has full descriptive statistics for participant characteristics.

Table 1 presents descriptive statistics for the study variables. The average levels of illness perceptions showed very strong timeline beliefs (*M* = 27.19, *SD* = 3.66), indicating that veterans, on average, perceived that their GWI would last a long time. Perceived consequences scores were also at the higher end of the range (*M* = 24.93, *SD* = 4.03), indicating high perceived severity of the consequences of GWI. Average levels of personal control beliefs and cyclic timeline beliefs were close to the mid-point of the possible range of scores, indicating moderate levels of perceived personal control over GWI and the degree to which GWI symptoms and experience varied from day to day/across time, respectively. Lastly, emotional representation scores were above the midpoint of the scale range (*M* = 20.81, *SD* = 3.32), indicating that veterans felt that GWI affected their emotions negatively (e.g., that GWI caused them anger, anxiety, and fear). Perceived concordance scores were, on average, above the scale range mid-point (*M* = 21.01, *SD* = 7.25), indicating generally high levels of concordance, but with a much higher degree of variability than in the illness perception scores.

Table 1 also presents the bivariate relationships between illness perceptions, perceived concordance, and outcomes (satisfaction, adherence, and disability). The largest relationships were seen between chronic timeline beliefs, consequences beliefs, and reported experience of disability from GWI (positive correlations); between chronic timeline beliefs, consequences beliefs and satisfaction immediately prior to the medical encounter (negative correlations); and between personal control beliefs and reported experience of disability (negative correlation). Multilevel analyses therefore controlled for veterans’ illness perceptions. In the tests of the main hypothesis, presented next, controlling for PTSD symptoms, depressive symptoms, and demographic factors did not alter the results in a meaningful way (in magnitude, valence, or significance of the fixed-effects estimates) for any of the outcomes; results are therefore presented without their inclusion as control variables.

### Satisfaction with care

**Figure 1.**
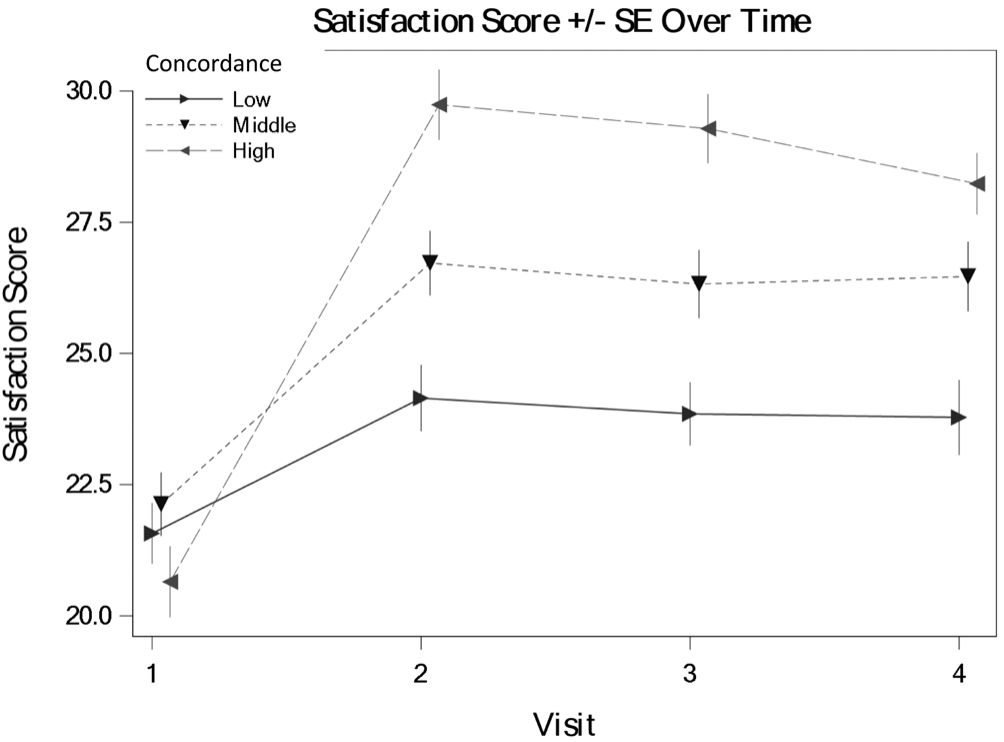
Interaction between perceived concordance level and time in predicting veterans’ satisfaction with care for their GWI. Note: The visits were immediately prior to the medical encounter (baseline = visit 1), 1 week after the medical encounter (visit 2 = 1 week), 1 month after the medical encounter (visit 3 = 1 month), and 6 months after the medical encounter (visit 4 = 6 months).

We tested the hypothesis that veterans with GWI who perceive greater concordance in illness perceptions with their provider (assessed immediately after the medical encounter) will report greater satisfaction with their care from that provider 1 week, 1 month, and 6 months after the encounter. The average level of satisfaction over all timepoints was 26.28 (*SE* = 3.71). There was not a statistically significant main effect of concordance on satisfaction (fixed-effect estimate = −0.01, *SE* = 0.05, *P* = .84). However, in support of the hypothesis, concordance interacted with time in predicting satisfaction, such that higher concordance was associated with greater satisfaction at all follow-up timepoints, compared to baseline (fixed-effect estimates = 0.36, *SE* = 0.06, *P* < .001 for 1-week follow-up; estimate = 0.36, *SE* = 0.06, *P* < .001 for 1-month follow-up; and estimate = 0.29, *SE* = 0.07, *P* < .001 for 6 months follow-up). Figure 1 is a graph of the interaction between concordance and time, predicting satisfaction. Full GEE results are available (link).

### Adherence to GWI care recommendations

**Figure 2.**
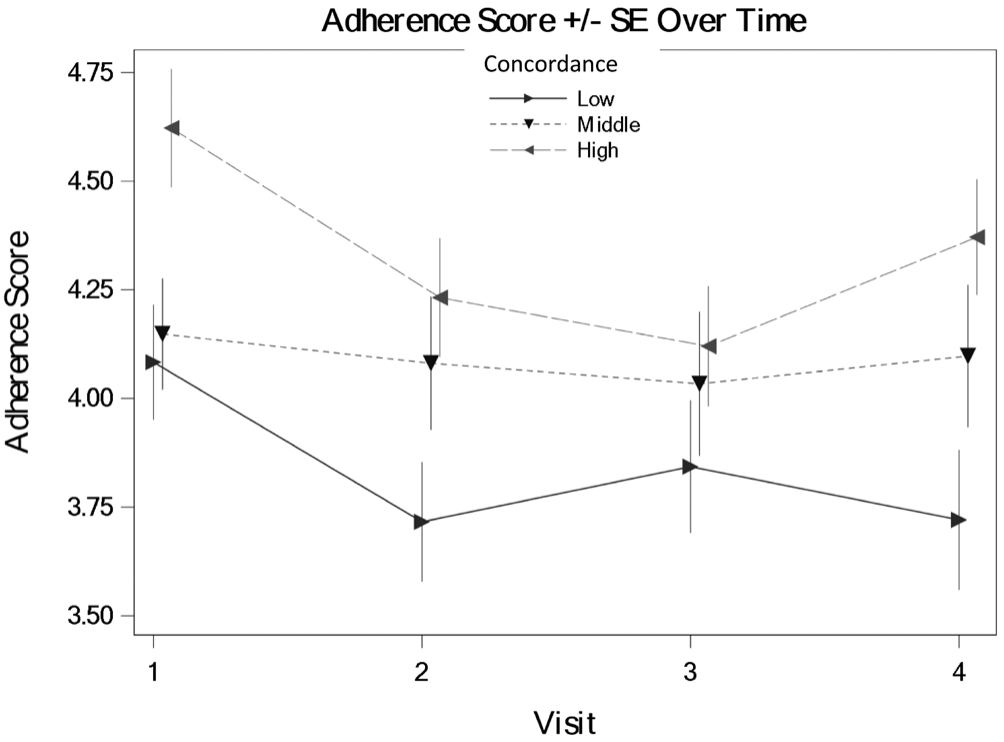
The main effect of perceived concordance on veterans’ reported GWI care plan adherence. Note: The visits were immediately prior to the medical encounter (baseline = visit 1), 1 week after the medical encounter (visit 2 = 1 week), 1 month after the medical encounter (visit 3 = 1 month), and 6 months after the medical encounter (visit 4 = 6 months).

We tested the hypothesis that veterans with GWI who perceive greater concordance in illness perceptions with their provider (assessed immediately after the medical encounter) will report greater adherence to the provider’s treatment care plans at 1 week, 1 month, and 6 months after the encounter. The average level of adherence to recommended treatment(s) by the provider over all timepoints and levels of concordance was 4.34 (*SE* = 0.74), and adherence was generally stable over time. Concordance was associated with adherence overall (fixed-effect estimate = 0.03, *SE* = 0.01, *P* = .03), such that higher levels of concordance were associated with greater adherence, as hypothesized. The relationship between concordance and adherence did not change with time; the interaction terms between time and concordance in predicting adherence were non-significant (estimates ranged from −0.01 to 0.01, *SE* = 0.01, *P*-values between .30 and .68). See Figure 2 for a graph of the main effect of concordance in predicting adherence. Full GEE results are available (link).

### Disability

**Figure 3.**
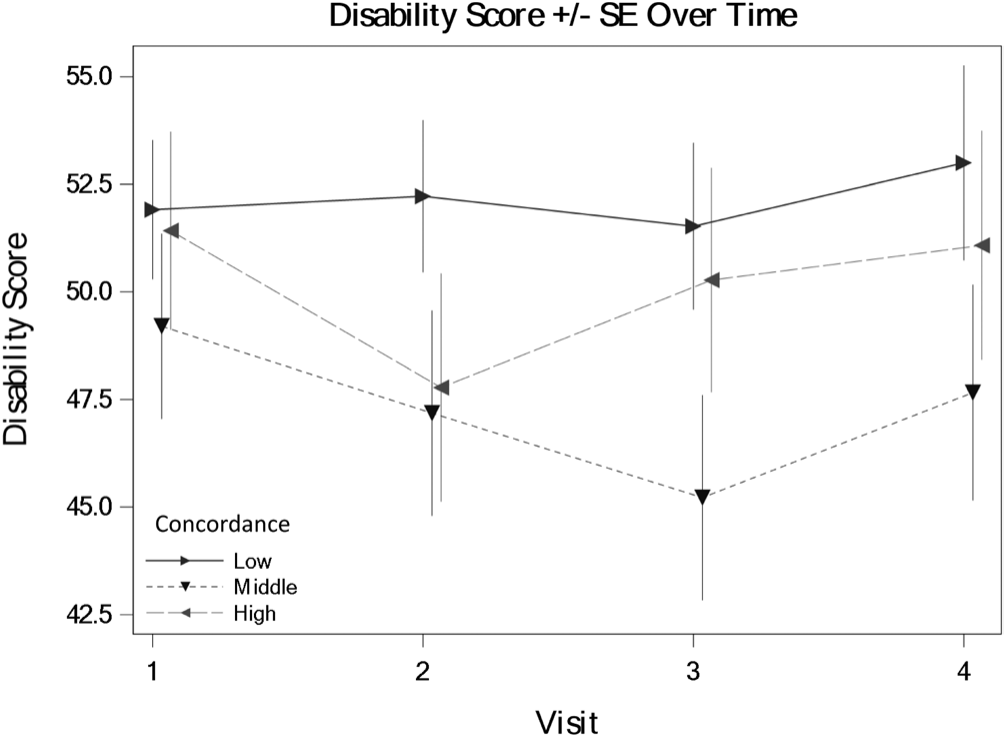
Perceived concordance did not predict differences in veterans’ reported disability levels. Note: The visits were immediately prior to the medical encounter (baseline = visit 1), 1 week after the medical encounter (visit 2 = 1 week), 1 month after the medical encounter (visit 3 = 1 month), and 6 months after the medical encounter (visit 4 = 6 months).

We tested the hypothesis that veterans with GWI who perceive greater concordance in illness perceptions with their provider (assessed immediately after the medical encounter) will report lower levels of disability at 1 week, 1 month, and 6 months after the encounter. The average level of disability over all timepoints and levels of concordance was 42.52 (*SE* = 14.07). There were no significant main effects of concordance or time on disability (fixed-effect estimate = −0.08, *SE* = 0.18, *P* = .64 for concordance across all timepoints; fixed-effect estimates for follow-up timepoints compared to baseline ranged from −2.45 to 2.18, *SE* ranging from 2.75 to 4.33, and *P-*values ranging from 0.43 to 0.67). Nor were there significant interaction effects between concordance and time in predicting disability (fixed-effect estimates for the interaction of concordance and each time point varies from −0.20 to 0.03, *SE* ranging from 0.12 to 0.19, and *P*-values ranging from 0.12 to 0.85). Figure 3 shows the general lack of support for the hypothesis in that concordance did not influence disability overall or over time. Full GEE results are available (link).

## Discussion

In general, we found support for the hypothesis that veterans’ perceptions of concordance with their provider regarding their GWI illness perceptions (i.e., perceived agreement with their provider regarding GWI diagnosis, associated symptoms, causes, timeline, control, and consequences) predicted their satisfaction with GWI care and adherence to GWI-related treatments over time. This was the case even after controlling for the (significant) relationships between the GWI perceptions and these outcomes.

Regarding satisfaction, even after controlling for veterans’ perceived consequences and perceived chronic timeline of GWI, which were associated with pre-visit levels of satisfaction, higher perceptions of concordance with the provider predicted significantly greater satisfaction with care at all subsequent timepoints. Since satisfaction is associated with continuity of care,^46^ perceptions of concordance may also promote continuity of care.

Regarding adherence to GWI treatment recommendations, although illness perceptions were unrelated to reports of adherence, concordance with the provider regarding illness perceptions was related to better adherence over time. These findings extend the evidence from existing literature that found perceived concordance was associated with greater satisfaction with care and adherence to treatment recommendations in a non-veteran, primary care setting,^28^ and in a cross-sectional study with military veterans.^29^

Although perceived concordance was not related to veterans’ reports of disability, the illness perceptions themselves were strongly related to disability, with the exception of cyclic timeline beliefs (beliefs that GWI is unpredictable, that GWI symptoms come and go). Existing literature has shown the possible benefits of providers eliciting the patients’ illness and treatment perceptions, to better understand and promote patients’ willingness to engage in and adhere to treatment recommendations.^28,29^ It may be that such communication behaviors could influence the veterans’ perceptions of their GWI and thereby their utilization of and adherence to the recommended treatment(s) for GWI, shifting disability levels over time. Veterans’ perceived concordance should match any shifts in perceptions that occur with discussion with the provider, though; it is, therefore, possible that perceived concordance may predict disability only at longer-term follow-ups than the 6 months observed in the current study.

The current study has several limitations and opportunities for future research. First, this study relied on veterans’ self-reports on measures, which is appropriate for constructs such as perceived concordance, illness perceptions, and satisfaction with care. However, associations between our study variables could have been influenced by common method variance. Future research could utilize objective measures of adherence and disability, and even longer-term follow-up assessments may be valuable, given the long durations seen with PPS and disability.^47^ Second, survey responses were acquired in multiple modalities—primarily via paper/hard copies through the postal mail but also over the phone as possible or by veteran preference—and were accepted any time until the next follow-up survey went out via postal mail. Although the delay in returning the surveys could have been due to barriers arising between completing the survey and posting it in the mail, it is possible that veterans delayed responding to the survey, beyond the intended follow-up timepoint. Therefore, the results cannot be interpreted by the exact timing of assessments following the baseline survey and medical visit; for example, we cannot make conclusions about the influence of concordance on outcomes within 1 week or 1 month; rather, each follow-up timepoint is a snapshot representation of a range of possible durations, with a maximum duration being near in time to the next assessment. Third, the results from our sample of veterans with GWI may not generalize to non-veteran populations with persistent physical symptoms or to other veterans with GWI who did not volunteer for the study. Fourth, there are potentially other influencing factors on both veterans’ perceptions of concordance and their reports of the outcomes that we did not capture in our measures and that are distinct from what we did measure and control for, such as depressive and PTSD symptoms. For example, the degree to which veterans experience demoralization could influence their illness and concordance perceptions and their reports of adherence and satisfaction with care.^48^ Future research can work to identify these factors and their importance for improving veterans’ care. Lastly, it is possible that a more granular view of GWI symptom experience over time and specific GWI treatments could provide a more complete picture of areas where concordance is particularly important for patient care. Specifically, regarding the experience of symptoms, some veterans may report flare-ups in their GWI symptoms, and these flare-ups could influence their adherence and disability levels in ways that our measures of timeline perceptions and outcomes were not able to capture. A more nuanced analysis, potentially with higher frequency data collection to capture episodes of flare-ups, could be used to evaluate any influence of those flare-ups compared to more chronic experiences of GWI. Regarding specific treatment types within GWI care plans, it may be that perceived concordance has a differential influence on adherence depending on the type of treatment; for example, ensuring concordance between patient and provider on the appropriateness of a highly accessible medication for treating GWI may be sufficient to greatly increase adherence, whereas concordance may not be sufficient to change adherence to a more resource-intensive or stigmatized treatment. The current study assessed adherence to “treatment care plans” in general, since GWI involves many different symptoms and even overlapping “syndromes,” such as chronic pain, chronic fatigue, and fibromyalgia, and their relevant treatments. The current aims were, therefore, best evaluated with a general adherence measure, in order for the items to be relevant to all respondents, a similar approach to other research on concordance of illness perceptions and treatment adherence.^28^

The current findings have direct implications for clinical care of PPS in general. Fostering concordance in the patient–provider relationship may boost satisfaction and treatment adherence, which are historically low among PPS populations. Qualitative work with the same study population has suggested ways that providers can foster perceived concordance in their patients regarding GWI by validating patients’ experiences of GWI, being more knowledgeable about GWI and up to date on GWI-specific treatment recommendations, and by earning patients’ trust through listening and good care coordination.^26^ Future research should focus on developing a model of care for veterans and other patients with PPS that includes elements designed to promote concordance between patients and providers on PPS and care recommendations.

## Data Availability

Due to the nature of this research, participants of this study did not agree for their data to be shared publicly, so supporting data are not available. Full results from the analyses are presented in supplemental material on the Open Science Framework, anonymized for review (OSF | Concordance of illness perceptions, Supplemental Material).

## Analytic code availability

The analytic code is available upon request.

## Study and analytic plan pre-registration

The study design, hypotheses, and analytic plan were pre-registered in the form of the funded grant proposal.
